# Kenneth Keown (1917-1985): Innovator in Early Cardiac Anesthesiology

**DOI:** 10.7759/cureus.67309

**Published:** 2024-08-20

**Authors:** Janette D McVey, Julie M Marshall, Quinn L Johnson, Frederick O'Donnell

**Affiliations:** 1 Department of Anesthesiology and Perioperative Medicine, University of Missouri School of Medicine, Columbia, USA

**Keywords:** cardiac arrhythmia, historical vignette, intraoperative hypothermia, mitral valve commissurotomy, cardiac anesthesiology

## Abstract

Kenneth Keown, MD, was a forward-thinking anesthesiologist who developed techniques to allow the safe practice of cardiac anesthesia and opened the door for the future development of more complex intracardiac surgical procedures. His early successful protocols for cardiac anesthesiology and his wide-reaching education of others on these methods earned him the designation of “the grand old man of anesthesia for inside-the-heart surgery” at a young age. His contributions also extended to groundbreaking research in hypothermia, lidocaine uses as an antiarrhythmic, and advocacy for anesthesiology as a specialty. We highlight the accomplishments of Dr. Keown that may be unrecognized by those outside the field of cardiac anesthesiology, as they paved the way for the success of modern cardiac surgery.

## Introduction and background

The goal of this article is to highlight not only the impact Dr. Kenneth K. Keown had on the field of cardiac anesthesiology, but also to acknowledge his accomplishments in educating others to advance intracardiac surgery options to those throughout the United States.

More than 65 years have passed since Dr. Keown published the first monograph on cardiac anesthesia, and it has been more than 75 years since his contributions helped launch the practice of anesthesia for intracardiac surgery. Yet, despite his contribution to establishing cardiac anesthesiology practices and advocating for the specialty, he may be unknown to those outside of this field. Dr. Keown was not just one of the first innovators of cardiac anesthesia but was also a leader and advocate of education in the field of anesthesiology.

## Review

Keown’s life and career

**Figure 1:**
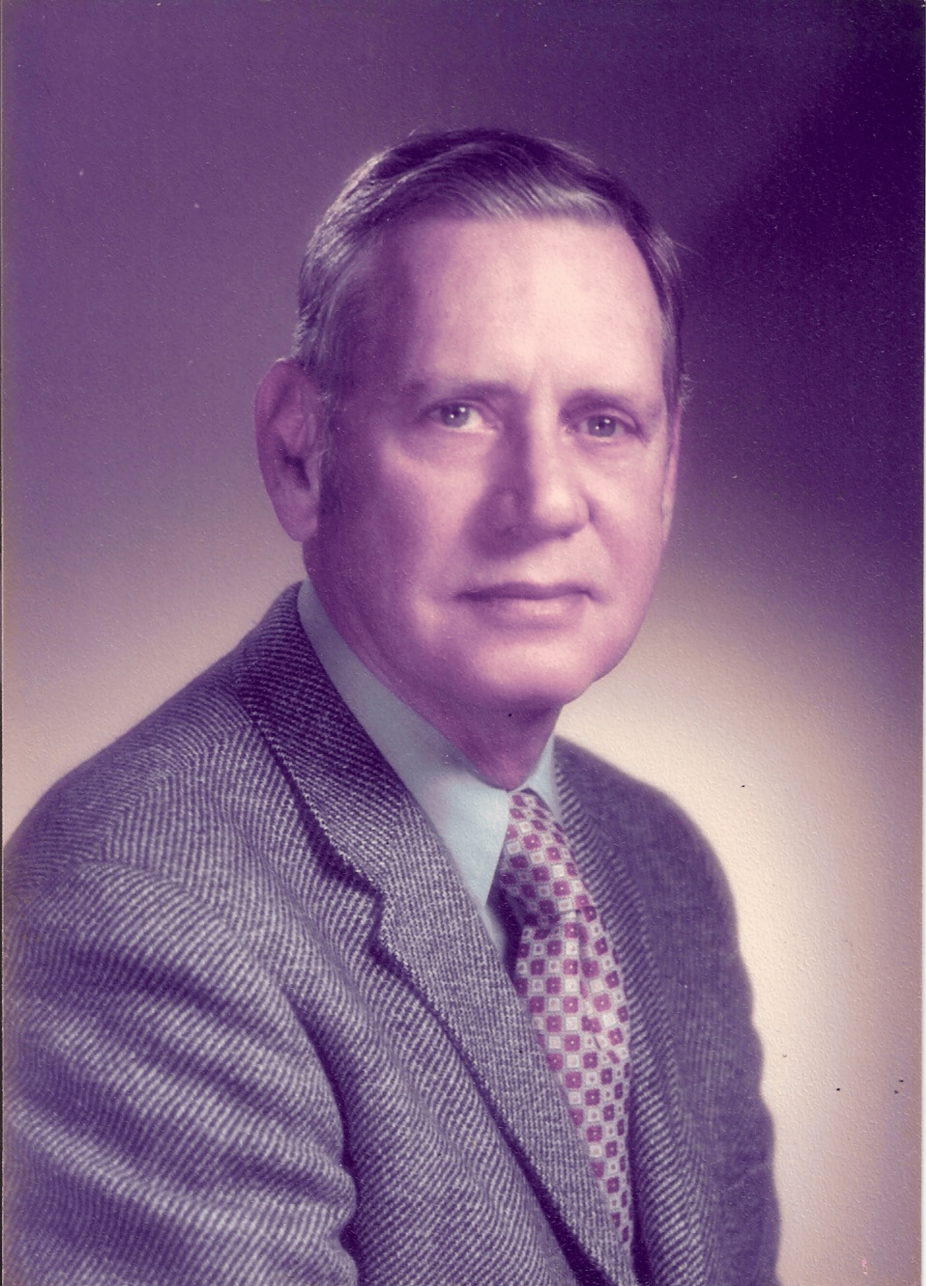
Kenneth K. Keown, circa 1974. Photo courtesy of Linda Keown, daughter of Dr. Keown. Used with permission.

Kenneth K. Keown started his life as the son of a general practitioner in Independence, Missouri (Figure 1). By accounts, his experience watching his father in his medical practice provided some initial direction for his future career. The senior Dr. Keown was interested in methods to provide pain relief during deliveries, which undoubtedly provided an early influence on the future anesthesiologist, Dr. Kenneth Keown [1,2]. Dr. Kenneth Keown began his training as a surgeon, completing medical school at Hahnemann Medical College of Philadelphia. Following an internship at Huron Road Hospital in Cleveland [1], Dr. Keown served in the US Army Medical Corp for four years. This military experience impressed upon him the need for physicians to be trained in the specialty of anesthesia [2]. Dr. Keown completed his two-year residency in anesthesiology at Hahnemann Hospital following his service in the US Army [2]. He remained at Hahnemann Hospital during his early career and during those years, he helped perfect anesthesia techniques for heart surgery that allowed for consistent, successful intracardiac surgery outcomes. It was also at Hahnemann Hospital that Dr. Keown published the first monograph on cardiac anesthesiology, *Anesthesiology for Surgery of the Heart* in 1956 [1-3].

Dr. Keown’s accomplishments were notable for the influence he brought to the subspecialty of cardiac anesthesiology, but he was also a strong advocate for leadership in medicine, medical education, and the establishment of anesthesiology as its own specialty [4]. As he returned to his home state of Missouri, Dr. Keown established the Department of Anesthesiology and served as chairman of that department until September of 1969 when he became Medical Director of the University of Missouri Medical Center [1]. During this period, he served in multiple leadership positions and advocated for nationwide training programs for physicians in anesthesiology as we began to care for complex patients undergoing more complex procedures [4].

History of early cardiac surgery and cardiac anesthesiology

Before the 1950s, cardiac surgery gained advances but met a large hurdle - safely providing anesthesia care to patients that would lead to successful outcomes. Anesthetic techniques at the time were very rudimentary, with ether and chloroform being the most used agents. Modern cardiac surgery began with operations on intrathoracic blood vessels rather than on the heart itself, beginning with a PDA (patent ductus arteriosus) ligation at Children’s Hospital of Boston in the late 1930s. In the years following, coarctation repair was performed in Stockholm, Sweden in 1944, and palliation of Tetralogy of Fallot in Baltimore, Maryland in 1944 [5]. In 1946, Merel Harmel and Austin Lamont described the anesthesia for the first one hundred Blalock-Taussig Operations (creation of an artificial shunt between one of the major systemic arteries and the pulmonary artery) and wrote the first cardiac anesthesiology manuscript [6]. The first instances of these anesthetics in pediatric patients describe endotracheal tubes fashioned out of large bore urethral catheters if intubation was needed, open drop ether anesthetics, and monitoring with only a finger on the carotid pulse. These anesthetics were further in partnership with nurse anesthetist Elizabeth Lank of Boston Children’s Hospital to include elective endotracheal intubation and cyclopropane anesthesia [5]. Even with these advancements, invasive surgery on the heart or its valves had not progressed due to fears of damage to the heart muscle, blood flow to the heart, or blood flow to the brain. In 1948, the first “closed” mitral commissurotomy was performed by Horace Smithy of the Medical University of South Carolina, followed quickly by the first closed aortic valvotomy in Philadelphia by Charles Bailey in 1950 [7]. In 1953, John Gibbon achieved the first successful use of a heart-lung machine for the repair of an atrial septal defect at Jefferson Medical College in Philadelphia [8].

Keown’s contributions to cardiac anesthesiology

The multitude of complications and risks associated with the prior stated procedures ushered in the field of cardiac anesthesiology and one of its many founders, Dr. Kenneth Keown, to implement protocols making intracardiac surgery an achievable enterprise. Advances had been made in the understanding of how anesthetic modalities affected the cardiovascular system, but techniques tailored to cardiac procedures were still in their early stages. Valvular diseases of the heart were common due to the endemic nature of rheumatic fever in the United States in the early 1900s [2]. Dr. Charles P. Bailey, Hahnemann’s Chief of Thoracic Surgery, worked closely with Dr. Keown, and together they developed the first methods to make cardiac surgery an acceptable option for patients with mitral valve disease. In June of 1948, they teamed up to perform an opening of the anterior and posterior commissures of a calcified mitral valve, a procedure from then on termed the “mitral valve commissurotomy” [2]. Keown advocated for light levels of anesthesia achieved with an endotracheal tube and nitrous oxide/oxygen mixture combined with 0.2% procaine intravenously followed by intermittent doses of sodium thiopental [9]. The goal was to have the patient awake and responsive at the end of the case to foster a smoother postoperative recovery and minimize cardiac depression and arrhythmia. Keown was also a proponent of proper surgical selection of patients for this surgery, emphasizing a detailed history and physical evaluation and frank discussion with patients regarding their chances of survival and lifestyle changes [2]. Mortality rates for cardiac surgery were high. Clinicians had to rely upon clinical judgment and experience as their early primary guides to patient selection and the anesthetic process.

**Figure 2:**
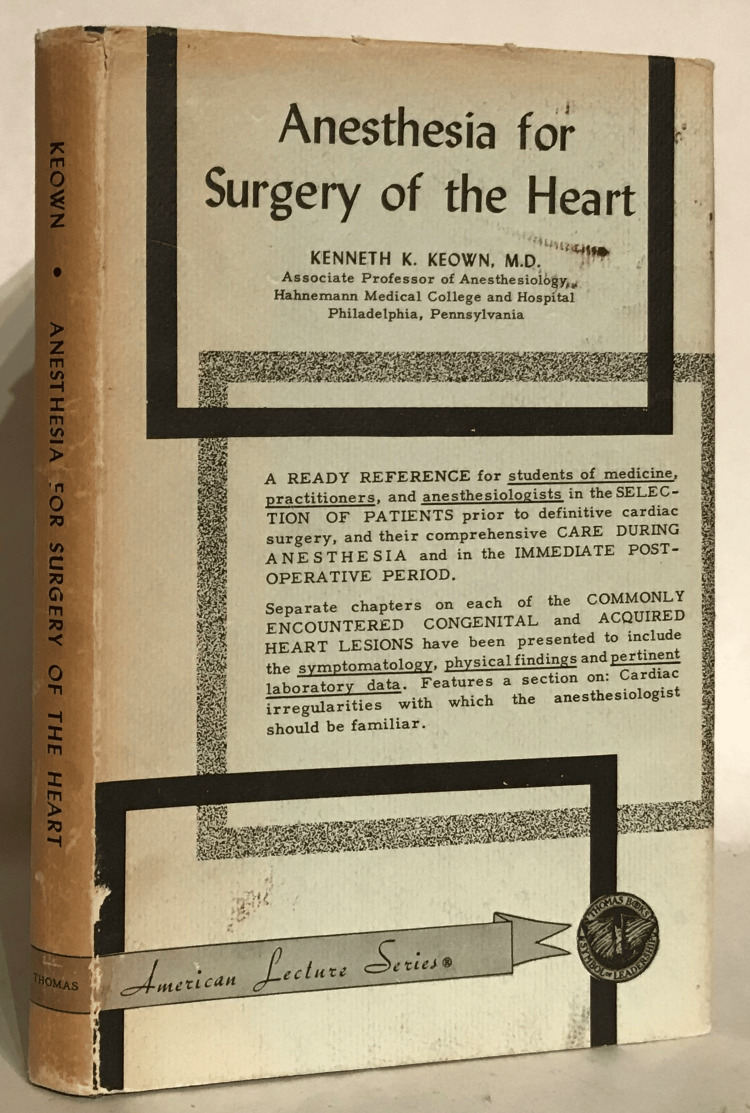
Anesthesia for Surgery of the Heart by Kenneth K. Keown, MD, 1st Edition (1956). Published by Charles C. Thomas, Springfield, Illinois. Photo courtesy of Thomas Dorn, Antiquarian Booksellers Association of America. Used with permission.

The success of Dr. Keown’s anesthetic technique and Dr. Bailey’s success in surgery attracted considerable media attention. This included an article in *Time* featuring Keown, describing him as “the grand old man of anesthesia for inside-the-heart surgery” at the ripe age of 36 [10]. Bailey subsequently achieved a cover of *Time* and attributed his success to Keown. In 1956, Keown published the textbook, *Anesthesia for Surgery of the Heart *(Figure 2), as a guide to cardiac anesthesia and advocated the importance of a team approach to the patient's surgical care. As noted in a review published in the journal *Anesthesiology*, “the author has probably given anesthesia to more patients for cardiac surgery than any other physician and so is eminently qualified to write this volume” [11]. Keown also shared his knowledge and expertise with other hospitals and colleagues who were expanding their cardiac surgery procedures, with many of those coming to him to observe procedures. He was widely regarded as a leading expert in anesthesiology for the following decades. Dr. Arthur Keats acknowledged his contributions in the 1983 American Society of Anesthesiologists *Rovenstine Lecture, Cardiovascular Anesthesia: Perceptions and Perspectives* stating “I also salute Kenneth Keown, not only for his extraordinary equanimity in facilitating the innovative operations of the colorful Charles Bailey, but for devising a method of anesthesia for patients with end-stage mitral valve disease that permitted the use of cautery” [12].

Development of induced hypothermia

Keown was additionally instrumental in developing hypothermia techniques specific to cardiac surgery. First introduced in 1941, induced hypothermia was not applied to cardiac surgery until 10 years later with few guidelines. The basic principles of hypothermia include decreasing cardiac irritability, minimizing stress factors through the anesthetic process, increasing oxygen availability, decreasing cerebral metabolism, and preserving cardiac function [13]. Dr. Keown’s original techniques were practiced through animal experiments and demonstrated that reduced body temperatures could be controlled, determined how long it could be maintained and outlined what complications to anticipate [13]. Keown’s earliest means of cooling was a “chill chest” (a household freezer) used during the closure of an atrial septal defect. As reported by his daughter, the original freezer used for this technique was obtained from their neighbor’s house and adapted for use at the hospital [14]. Later techniques employed various means of ice water baths or circulating cooling blankets [2]. The increased metabolic demand of shivering was abated by thiopental and/or neuromuscular blockade. Electrophysiology, respiratory function, and blood pressure were all observed, and rewarming protocols were developed. These early studies were vital to the hypothermic techniques employed today.

Use of lidocaine as an antiarrhythmic

Although it had long been recognized that intraoperative correction of cardiac arrhythmias was essential to maintaining homeostasis during surgery, Keown’s early work with lidocaine established its safe, effective use as an antiarrhythmic agent that could be dosed repeatedly to terminate significant dysrhythmias without negatively affecting cardiac function [15]. Nearly 65 years since Keown’s experience with over 500 cases using lidocaine, it is still a first-line treatment for arrhythmia in the intraoperative period.

Founding the Department of Anesthesiology at the University of Missouri

In September of 1956, the University of Missouri opened a new hospital in Columbia, Missouri. Dr. Keown arrived a year after the hospital opened and was the first chief of anesthesiology as a division in the Department of Surgery. His previous pioneering work at Hahnemann Medical College made him the obvious choice to help establish an open-heart surgery program in Columbia, Missouri [16]. When he arrived, Dr. Keown was the first and only anesthesiologist in Columbia and one of only 71 total anesthesiologists in the state of Missouri [17]. He established an anesthesiology residency program at the University of Missouri [1]. In addition to the typical duties of an anesthesiologist [17], Dr. Keown also was instrumental in initiating and rapidly expanding a respiratory therapy service and training program. Because of anesthesiologists' unique training and expertise, they were solely responsible for the administration, prescription, and delivery of oxygen to patients. Recognizing the critical need to utilize oxygen more frequently to a growing patient population but lacking adequate personnel, Dr. Keown created a school of respiratory therapy as one of the first bachelorette programs in Inhalational Therapy in the nation [18], oversaw the development of a curriculum, and eventually helped establish a master’s degree program in respiratory therapy at the University of Missouri [17].

On July 1, 1966, the University formally created the Department of Anesthesiology with Dr. Keown named as the first Chair [16]. The creation of a separate department was not easily obtained and required years of advocating, educating, and demonstrating a need for anesthesia to separate from the Department of Surgery. His impact expanded beyond just the University of Missouri as three of the junior faculty that he recruited and mentored eventually left to become chairs of anesthesia at the University of Pennsylvania, Louisiana State University, and Tennessee-Knoxville. Of course, he mentored the succeeding chair at the University of Missouri who also became a future president of the American Society of Anesthesiology [17]. In 1969, Dr. Keown stepped down as department chair and accepted the position of Medical Director of the University Medical Center. He remained in that role for six years and chose to return to the Department of Anesthesiology to continue his primary mission of teaching and training the next generation of doctors and anesthesiologists [16]. Dr. Keown continued to serve in leadership roles in the American Medical Association, the American Society of Anesthesiologists, the Missouri Society of Anesthesiologists, the Academy of Anesthesiology, and the International Anesthesia Research Society [1,4].

## Conclusions

Kenneth Keown, MD, created clinical protocols that allowed reproducible, safe anesthetic care for patients undergoing mitral valve commissurotomy; however, his influence extends significantly beyond this procedure. As he educated the growing national community of cardiac anesthesiologists, he provided a path forward for creating techniques to keep patients safe in this growing field. With the increasing number of cardiac procedures performed each year in the United States to improve the lives of our patients, we owe Dr. Keown credit and gratitude for his contribution to this area of medicine.
